# Computational Comparative Study of Tuberculosis Proteomes Using a Model Learned from Signal Peptide Structures

**DOI:** 10.1371/journal.pone.0035018

**Published:** 2012-04-09

**Authors:** Jhih-Siang Lai, Cheng-Wei Cheng, Ting-Yi Sung, Wen-Lian Hsu

**Affiliations:** Institute of Information Science, Academia Sinica, Taipei, Taiwan; University of South Florida, United States of America

## Abstract

Secretome analysis is important in pathogen studies. A fundamental and convenient way to identify secreted proteins is to first predict signal peptides, which are essential for protein secretion. However, signal peptides are highly complex functional sequences that are easily confused with transmembrane domains. Such confusion would obviously affect the discovery of secreted proteins. Transmembrane proteins are important drug targets, but very few transmembrane protein structures have been determined experimentally; hence, prediction of the structures is essential. In the field of structure prediction, researchers do not make assumptions about organisms, so there is a need for a general signal peptide predictor.

To improve signal peptide prediction without prior knowledge of the associated organisms, we present a machine-learning method, called SVMSignal, which uses biochemical properties as features, as well as features acquired from a novel encoding, to capture biochemical profile patterns for learning the structures of signal peptides directly.

We tested SVMSignal and five popular methods on two benchmark datasets from the SPdb and UniProt/Swiss-Prot databases, respectively. Although SVMSignal was trained on an old dataset, it performed well, and the results demonstrate that learning the structures of signal peptides directly is a promising approach. We also utilized SVMSignal to analyze proteomes in the entire HAMAP microbial database. Finally, we conducted a comparative study of secretome analysis on seven tuberculosis-related strains selected from the HAMAP database. We identified ten potential secreted proteins, two of which are drug resistant and four are potential transmembrane proteins.

SVMSignal is publicly available at http://bio-cluster.iis.sinica.edu.tw/SVMSignal. It provides user-friendly interfaces and visualizations, and the prediction results are available for download.

## Introduction

Signal peptides are short sequences that start from the N-terminus and control protein secretion. They are related to drug targets, protein production, and even biomarker discovery [1]–[4]. Normally, signal peptides in proteins are recognized and cleaved by their corresponding proteases, and then the cleaved proteins are secreted [5]. In some cases, however, instead of being cleaved, the signal peptides form signal anchors, which are a type of transmembrane protein [6]. Moreover, as shown by Gierasch [7] signal peptides are interchangeable as well as highly tolerant, i.e., they allow some mutations. Thus it is important to identify signal peptides in proteins.

Proteins targeting to organelles or outside of the cell sometimes need a cleavable signal peptide. Signal peptide has its systematic structure, an amino-terminal positively charged region (n-region), followed by a central, hydrophobic region (h-region), then followed by a more polar carboxy-terminal region (c-region) [6]. The hydrophobic core of h-region could be recognized by the SRP (signal recognition particle). C-region usually contains a motif before the cleavage site that can be cleaved by appropriate protease. For example, the bacterial signal peptides consist of positive charge residues, hydrophobic core, and a motif such as Ala-X-Ala, just before the cleavage site to direct the protein going through the Sec pathway. The Tat signal peptide also has the above (n, h, c)-regions structure, particularly having consecutive arginines in n-region, to direct the protein going through the Tat pathway. The lipoprotein signal peptide also has the above structure, and particularly a cysteine follows the cleavage site for lipid modification.

Modifying the residues of these cleavable signal peptides may affect protein secretion. For example, the secretion efficiency may be mediated by hydrophobicity in h-region and charge in n-region [8], [9]. Modification at cleavage site may extend or shorten the mature protein sequence, and then may slightly alter the protein structure. Completely removing c-region may yield the signal peptide uncleaved and form a signal anchor for transmembrane proteins.

Since the pairwise sequence similarity of signal peptides is usually low, they cannot be detected simply by sequence alignment analysis [10]. To predict signal peptides, rules have been devised for the analysis of signal peptide cleavage sites [11]. Combined the rules with the signal peptide structure, i.e., (n, h, c)-regions, can lead to accurate signal peptide prediction. However, a major difficulty with this technique is that signal peptides may be misclassified as transmembrane domains, and vice versa, because both regions contain hydrophobic cores, and hydrophobicity is a key feature of signal peptide prediction methods [6]. Transmembrane proteins are important drug targets, but very few transmembrane protein structures have been determined experimentally. Accurate prediction of transmembrane protein structures is essential. If the transmembrane domain of a transmembrane protein is misclassified as a signal peptide, or vice versa, it would lead to incorrect transmembrane protein structure prediction and also inaccurate secretion analysis. Several methods have been developed for signal peptide prediction based on three domains of a signal peptide structure. For example, SignalP uses neural networks and hidden Markov models to construct the (n, h, c)-regions and improve the disambiguation between transmembrane proteins and signal peptides [12]–[14]; and PrediSi exploits a position weight matrix to predict cleavage sites [15]. Phobius, which uses a hidden Markov model, was the first predictor to predict both the topologies of transmembrane proteins and signal peptides [16]. RPSP is a neural network-based method designed for proteomic analysis [17]; and Philius uses dynamic Bayesian networks to model transmembrane protein topology and signal peptide [18].

Although the structure of (n, h, c)-regions provides good clues for signal peptide prediction and clearer rules have been defined for the c-region, the n-region and h-region have ambiguous boundaries and are diversified in terms of sequences and organisms. Thus in this paper we present a machine learning approach based on support vector machines (SVMs), called SVMSignal, to learn the structures of signal peptides and classify signal peptides from transmembrane proteins. SVMSignal uses basic biochemical profiles as features and also defines a novel feature to capture the inter-profile relationships that describe the structural properties of signal sequences.

We compared the performance of SVMSignal with that of existing methods on two benchmark datasets, the signal peptide database SPdb [19] and a hybrid dataset compiled from UniProt/Swiss-Prot [20] and PDBTM [21] (Protein Data Bank of Transmembrane Proteins), a transmembrane protein structure database. We used experimentally determined signal peptide sequences from SPdb and UniProt/Swiss-Prot as the signal peptide benchmark to demonstrate the sensitivities of various signal peptide predictors. In order to evaluate the specificities and classification abilities of different predictors, we collected soluble proteins from UniProt/Swiss-Prot and transmembrane proteins from both UniProt/Swiss-Prot and PDBTM as benchmark. Our method SVMSignal achieved good performance on the benchmark datasets.

Signal peptides are crucial to protein secretion, and secretome analysis is important in pathogen studies because it has been shown that some hosts are affected by proteins secreted from bacteria [22], [23]. There are at least six known secretion systems, i.e., Types I to VI, as well as the Sec and Tat pathways in Gram-negative bacteria [5]. Signal peptides are not responsible for all of the secretions. Although proteins containing signal peptides (called signal peptide proteins hereafter) cannot characterize the full secretome, it is still necessary to use sequence information to discover potential secreted proteins for further research [24], [25].

HAMAP (High-quality Automated and Manual Annotation of microbial Proteomes) microbial database [26] provides 1292 curated microbial proteomes and description of microbial pathogens, and proteomes in the database are also integrated with UniProt/Swiss-Prot. It is considered as an important database for pathogen studies [27], [28]. We applied our method to HAMAP to predict signal peptides in proteomes so that signal peptide-dependent secreted proteins could be identified.

From the pathogens recorded in the HAMAP database, we selected tuberculosis to conduct secretome analysis because it is a historical human interactive pathogen, and it is still difficult to treat in some cases, e.g., drug-resistant strains and immunocompromised HIV patients. Recently, Walzl et al. presented a review paper that suggests using an “omics" approach to find potential immunological biomarkers of tuberculosis [29]. To this end, we made an in-depth analysis of proteins with signal peptides in Mycobacterium tuberculosis and Mycobacterium bovis, which can cause tuberculosis in humans and cattle, respectively. It is also possible for Mycobacterium bovis to infect humans via certain foods, such as unpasteurized milk. We selected seven strains from the two pathogens for study, including virulent strains, attenuated strain, and drug-resistant strains. Due to their close lineage, the comparative study of signal peptide-dependent secreted proteins belonging to different strains can provide clues about the tuberculosis mechanism, virulence factors or biomarkers. The signal peptide prediction results of the entire HAMAP database are provided for further research.

## Results

### Performance evaluation of SVMSignal

We compared the performance of SVMSignal with that of five existing predictors, namely, Phobius [16], RPSP [17], Philius [18], SignalP [12]–[14] (specifically SignalP 3.0), and PrediSi [15]. Each of the last two predictors provides three models specifically developed for Gram-positive bacteria, Gram-negative bacteria and eukaryotes. We included all of the models in the performance evaluation.

To avoid over-estimating SVMSignal's performance in comparison with the other predictors, we used the original Phobius 2004 dataset to train our model. We tested all of the methods on the two benchmark datasets mentioned earlier, i.e., the signal peptide database SPdb [19] (called the SPDB dataset hereafter), and a hybrid dataset compiled from UniProt/Swiss-Prot [20] and PDBTM [21] in 2010. Note that we filtered each of the benchmark datasets by removing sequences with over 30% similarity to sequences in the training dataset and within the dataset itself. Both datasets were decomposed by organism into mixed, eukaryotes, and bacteria for analysis; archaea and viruses were omitted because there was insufficient data for them. We further decomposed the second benchmark dataset into SP (proteins with signal peptides), TM (proteins with transmembrane domains only), and G (proteins without signal peptides or transmembrane domains) datasets. Note that the SP dataset contains very few proteins with both transmembrane domains and signal peptides. All of the above datasets are provided in the Supporting Information (Dataset S1).

To evaluate the performance of various predictors, we define sequences with signal peptides as positive (P) data, and those without signal peptides as negative data (N). We use the following metrics to evaluate the performance 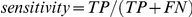, 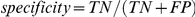 and 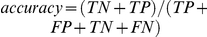, where T and F denote “true" and “false", respectively. Accuracy alone is insufficient to reflect a global perspective of good sensitivity in signal peptides and specificity in non-signal peptides, especially specificity in transmembrane domains required by a good predictor, due to the data imbalance. Therefore, to evaluate the performance from a global perspective, we use MCC (Matthew's correlation coefficient), which is formulated as 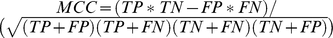.

### Evaluating SVMSignal's ability to disambiguate proteins with and without signal peptides

Predictors sometimes confuse signal peptides with transmembrane domains due to hydrophobic composition. We evaluated SVMSignal's ability to classify signal peptides and non-signal peptides, i.e., to disambiguate the SP dataset from the TM and TM+G datasets, denoted by SP/TM and SP/(TM+G), respectively. The MCCs of SVMSignal and the other predictors on different organism datasets are detailed in Table 1. SVMSignal outperformed the other predictors on the mixed organisms and eukaryotes in SP/TM and SP/(TM+G), except on the eukaryotes in SP/TM as the second best, with 2.24% slightly worse than the best SignalP model. Notably, in the mixed organisms, SVMSignal outperformed the second best predictor by 0.54% and 3.31% on SP/TM and SP/(TM+G), respectively. It also achieved the second best performance on the bacteria in SP/TM and the third best performance on the bacteria in SP/(TM+G).

**Table 1:** MCCs (%) of various predictors on classification of proteins with and without signal peptides.

Species	Data set	Cleavable	SVMSignal	Phobius	Philius	RPSP	SignalP NN+Euk	SignalP HMM+Euk	SignalP NN,G+	SignalP HMM,G+	SignalP NN,G−	SignalP HMM,G−	PrediSi Euk	PrediSi G+	PrediSi G−
Mixed	SP/TM	12.83%	**66.24%**	65.70%	64.36%	33.23%	42.22%	57.12%	42.88%	28.42%	46.54%	37.98%	20.96%	15.53%	18.73%
	SP/(TM+G)	32.73%	**86.25%**	82.94%	76.93%	82.21%	81.03%	78.65%	63.08%	71.21%	64.90%	75.75%	77.07%	54.51%	58.35%
Euk	SP/TM	12.68%	58.11%	54.91%	46.60%	39.09%	**60.35%**	48.79%	22.97%	19.58%	29.38%	26.71%	28.30%	6.24%	6.38%
	SP/(TM+G)	35.48%	**87.83%**	83.13%	75.34%	86.53%	84.54%	78.15%	56.47%	63.87%	58.80%	70.72%	80.31%	45.17%	50.18%
Bac	SP/TM	9.81%	72.14%	71.80%	**75.05%**	27.79%	26.11%	59.73%	65.18%	59.78%	64.14%	66.21%	11.51%	40.06%	47.02%
	SP/(TM+G)	29.57%	84.72%	83.92%	80.76%	74.45%	75.34%	80.57%	79.17%	**86.07%**	79.95%	85.89%	71.13%	72.97%	73.81%

As shown in Table 2, the accuracy of SVMSignal's classification with respect to different organisms ranged from 90% to 92% for SP/TM and exceeded 96% for SP/(TM+G). SVMSignal achieved the best accuracy in classifying SP/(TM+G) of the mixed organisms and eukaryote datasets. It achieved the second best accuracy in SP/TM of mixed organisms and SP/(TM+G) of bacteria with at most 0.4% difference to the best accuracy, and the third best accuracy in the remaining datasets with at most 2% gap from the best accuracy.

**Table 2:** Accuracies (%) of various predictors on classification of proteins with and without signal peptides.

Species	Data set	Cleavable	SVMSignal	Phobius	Philius	RPSP	SignalP NN+Euk	SignalP HMM+Euk	SignalP NN,G+	SignalP HMM,G+	SignalP NN,G−	SignalP HMM,G−	PrediSi Euk	PrediSi G+	PrediSi G−
Mixed	SP/TM	88.56%	91.14%	**91.54%**	90.85%	78.61%	87.56%	89.85%	82.79%	68.36%	85.07%	77.11%	81.29%	56.82%	63.98%
	SP/(TM+G)	56.43%	**96.72%**	95.71%	93.80%	96.04%	95.18%	94.38%	89.28%	93.77%	89.64%	94.51%	94.30%	90.12%	90.40%
Euk	SP/TM	94.05%	92.02%	92.33%	90.77%	82.94%	**94.05%**	92.18%	79.81%	58.69%	83.10%	70.89%	86.85%	46.64%	55.40%
	SP/(TM+G)	57.71%	**96.79%**	95.26%	92.48%	96.64%	95.61%	93.41%	85.46%	91.53%	85.99%	92.78%	94.61%	87.14%	87.54%
Bac	SP/TM	79.82%	90.21%	90.50%	**91.10%**	71.51%	76.56%	86.05%	88.43%	86.94%	88.43%	89.32%	72.11%	75.67%	80.42%
	SP/(TM+G)	53.66%	96.38%	96.10%	95.20%	94.39%	94.01%	95.34%	94.72%	**96.76%**	94.91%	96.62%	92.86%	93.86%	93.67%

### Sensitivity and Specificity of SVMSignal's predictions on the signal peptide benchmark datasets

The sensitivities of SVMSignal and the other predictors on the signal peptide benchmark datasets, SPDB and SP, are shown in Table 3. Although SVMSignal was trained on an old dataset and it was not trained on different organisms, it performed well compared to most of the predictors. As SignalP was trained specifically on different organisms, reflected by variations in the sensitivities of different models, SVMSignal performed slightly inferior to SignalP by at most 0.92% on the mixed organism datasets, at most 2.88 on the eukaryotes datasets, and at most 2.58% on the bacteria datasets.

**Table 3:** Sensitivities (%) of various predictors on the signal peptide protein benchmark datasets.

Species	Dataset	Cleavable	SVMSignal	Phobius	Philius	RPSP	SignalP NN+Euk	SignalP HMM+Euk	SignalP NN,G+	SignalP HMM,G+	SignalP NN,G−	SignalP HMM,G−	PrediSi Euk	PrediSi G+	PrediSi G−
Mixed	SPDB	99.24%	95.27%	93.90%	94.05%	85.82%	**96.19%**	96.04%	80.34%	58.38%	84.76%	67.53%	91.46%	49.54%	58.38%
	SP	97.67%	91.12%	**92.01%**	91.12%	79.91%	91.12%	91.34%	83.68%	67.26%	86.24%	76.80%	86.13%	55.27%	63.71%
Euk	SPDB	100.00%	95.97%	94.43%	94.43%	85.99%	**98.85%**	97.50%	76.97%	50.48%	82.15%	61.61%	92.71%	42.80%	52.98%
	SP	98.52%	91.76%	92.59%	91.43%	82.54%	**94.40%**	93.08%	80.72%	57.17%	83.86%	70.02%	88.47%	45.47%	55.19%
Bac	SPDB	96.55%	93.97%	92.24%	93.10%	84.48%	86.21%	90.52%	95.69%	93.10%	**96.55%**	94.83%	87.07%	80.17%	82.76%
	SP	95.99%	90.51%	91.61%	90.88%	74.45%	84.31%	87.96%	90.51%	90.15%	91.24%	**92.34%**	81.75%	76.64%	82.48%

We compared the specificities of SVMSignal and the other predictors on proteins without signal peptides in the TM and TM+G datasets. As shown in Table 4, for the TM classification, SVMSignal achieved the best performance on the mixed organism and eukaryote datasets, and was only outperformed by Philius on the bacteria dataset. For the TM+G classification, SVMSignal's performance differed from the best performance by at most 1.48%; however, on the three organism datasets of TM+G, it achieved over 97% specificity, which was very close to the specificity achieved by SignalP.

**Table 4:** Specificities (%) of various predictors on the non-signal peptide protein benchmark datasets.

Species	Data set	Cleavable	SVMSignal	Phobius	Philius	RPSP	SignalP NN+Euk	SignalP HMM+Euk	SignalP NN,G+	SignalP HMM,G+	SignalP NN,G−	SignalP HMM,G−	PrediSi Euk	PrediSi G+	PrediSi G−
Mixed	TM	9.62%	**91.35%**	87.50%	88.46%	67.31%	56.73%	76.92%	75.00%	77.88%	75.00%	79.81%	39.42%	70.19%	66.35%
	TM+G	50.09%	97.58%	96.28%	94.21%	**98.52%**	95.80%	94.85%	90.13%	97.85%	90.17%	97.24%	95.56%	95.48%	94.50%
Euk	TM	9.38%	**96.88%**	87.50%	78.13%	90.63%	87.50%	75.00%	62.50%	87.50%	68.75%	87.50%	56.25%	68.75%	59.38%
	TM+G	50.38%	97.69%	95.74%	92.67%	**99.17%**	95.83%	93.47%	86.31%	97.69%	86.37%	96.87%	95.71%	94.62%	93.35%
Bac	TM	9.52%	88.89%	85.71%	**92.06%**	58.73%	42.86%	77.78%	79.37%	73.02%	76.19%	76.19%	30.16%	71.43%	71.43%
	TM+G	47.32%	97.26%	96.77%	95.84%	97.37%	95.46%	96.44%	95.35%	**97.76%**	95.46%	97.26%	94.53%	96.44%	95.35%

### Application of SVMSignal to tuberculosis pathogen study

We applied signal peptide prediction to secretome analysis in tuberculosis pathogen since tuberculosis is a well-known infectious disease that can be fatal. Notably, signal peptides of tuberculosis proteomes are related to its virulence [30]–[32]. Since very few proteins of tuberculosis strains have been annotated, we used SVMSignal to predict signal peptides and performed a comparative study of the predicted signal peptide proteins of tuberculosis strains. Our objective was to identify some critical proteins that would be worth further investigation.

For our study, we selected seven interesting strains related to tuberculosis: four strains from Mycobacterium tuberculosis and three strains from Mycobacterium bovis in the HAMAP database [26]. Specifically, the four strains of Mycobacterium tuberculosis are MYCTU, MYCTA, MYCTF, MYCTK, where MYCTU is the virulent H37Rv strain, MYCTA is the highly attenuated H37Ra strain, MYCTF is the virulent strain family 11, and MYCTK is the drug resistant KZN 1435 strain. The other three strains are one strain of Mycobacterium bovis coded as MYCBO, and two strains of Mycobacterium bovis bacillus Calmette–Guérin (BCG) coded as MYCBP and MYCBT, respectively. MYCBP is a BCG strain developed at the Pasteur Institute in Paris and MYCBT is another BCG strain used in Japan. In the following discussion, we use the species code instead of the full name to represent each of the strains and the UniProt/Swiss-Prot accession number to represent a protein.

To identify signal peptide proteins that characterize each pathogen strain, we first applied SVMSignal to the seven proteomes of the strains, and we were only interested in the *predicted* signal peptide proteins. The statistics of the signal peptide proteins in the seven proteomes, i.e., four strains from Mycobacterium tuberculosis and three strains from Mycobacterium bovis, are shown in Table 5. There are 334 to 345 signal peptide proteins, i.e., 8.33% to 8.73% of the total proteins, in each proteome. Signal peptide proteins predicted to contain transmembrane domains account for 1.59% to 1.88% of the proteins in a proteome. Furthermore, the number of multi-spanning transmembrane proteins is double that of single-spanning transmembrane proteins. The average length of signal peptides is 28 to 29 residues, and the average length of the entire bacteria pathogen in the HAMAP database is 26.02. See the Discussion section and Supporting Information (Datasets S2 and S3) for more details.

**Table 5:** Basic statistics in the selected seven proteomes of Mycobacterium tuberculosis (*) and Mycobacterium bovis (**).

code	name	# proteins	# SP proteins (%)	SP mean length	# SPTM proteins (%)	# SPTM single	# SPTM multi
MYCTU	*Mycobacterium tuberculosis	3950	345 (8.73%)	28.4	71 (1.80%)	23	48
MYCTA	*strain ATCC 25177/H37Ra	3990	345 (8.65%)	28.2	69 (1.73%)	22	47
MYCTF	*strain F11	3905	334 (8.55%)	29.0	66 (1.69%)	22	44
MYCTK	*strain KZN 1435/MDR	4024	335 (8.33%)	28.7	64 (1.59%)	22	42
MYCBO	**Mycobacterium bovis	3910	334 (8.54%)	28.2	72 (1.84%)	22	50
MYCBP	**strain BCG/Pasteur 1173P2	3891	336 (8.64%)	28.1	73 (1.88%)	25	48
MYCBT	**strain BCG/Tokyo 172/ATCC 35737/TMC 1019	3906	340 (8.70%)	28.2	73 (1.87%)	25	48

### Comparative Study on the selected tuberculosis strains

To compare the seven tuberculosis strains, we used CD-HIT [33] and the 30% similarity threshold to cluster the signal peptide proteins by their sequences with signal peptides removed, i.e., their secreted sequences. Since secreted proteins affect their hosts and are of interest to us, we used them to cluster the corresponding signal peptide proteins. The process generated 313 clusters, of which 10 contained only one protein, whose similarity to all the other sequences was less than 30%. We call such a signal peptide protein a *unique protein* because it does not occur in any of the other six proteomes. Table 6 lists all the unique proteins found in the seven proteomes. MYCBP and MYCBT do not contain any unique proteins, while each of the other five strains has at least one unique protein. Interestingly, the human tuberculosis drug resistant strain MYCTK has six unique proteins. Furthermore, we removed signal peptides from the unique proteins and used TMHMM [34], [35] to predict whether their secreted sequences contain transmembrane domains. Four of the unique proteins, O06239, A5WU15, C6DWG6 and Q7U0W0, were predicted as multi-spanning transmembrane proteins and also annotated as transmembrane proteins in the UniProt/Swiss-Prot database.

**Table 6:** List of unique proteins and their similar non-signal peptide proteins.

Unique proteins					Similar proteins					
Species	ID	gene	TMHMM results	Seq. length(after cleaved)	Species	ID	gene	SVMSignal results	Seq. length	type (similar protein)
MYCTU	O06239	**Rv2136c**	multi-pass TM	282 (261)	MYCTK	C6DPS4	TBMG_01845	non SP	276	residues missing
MYCTA	A5U3R8	MRA_1910	non TM	343(283)	MYCTU	O07733	**Rv1899c**	non SP	359	residues addition
MYCTF	A5WU15	TBFG_13829	multi-pass TM	1082(1051)	MYCTU	P72030	**Rv3795**	non SP	1098	residues addition
MYCTK	C6DL40	TBMG_03351	non TM	481(457)	MYCTU	O53355	**Rv3303c**	non SP	493	residues missing
MYCTK	C6DUE9	TBMG_02759	non TM	362(329)	MYCTU	O06291	**Rv1223**	non SP	528	residues addition
MYCTK	C6DWG6	TBMG_03065	multi-pass TM	262(239)	MYCTU	O05916	**Rv0924c**	non SP	428	residues addition
MYCTK	C6DSD8	TBMG_00349	non TM	138(120)	MYCTU	O06296	Rv0345	non SP	136	residues missing
MYCTK	C6DTY5	TBMG_00617	non TM	111(89)	MYCTA	A5TZZ2	MRA_0618	non SP	139	residues addition
MYCTK	C6DQS5	TBMG_03974	non TM	53(28)	-	-		-		
MYCBO	Q7U0W0	Mb1023	multi-pass TM	358(330)	MYCBT	C1ALY5	JTY_1023	non SP	358	residue replacement
MYCBP	-		-		-	-		-	-	-
MYCBT	-		-	-	-	-		-	-	-

Next, for each unique protein, we used BLAST to search against the seven proteomes for similar proteins and the results are shown in Table 6. Note that the homologous protein of each unique protein is not signal peptide protein. The alignment results of the homologous protein pairs are provided in Supporting Information (Text S1). Six of the homologous protein pairs share a very high sequence similarity (over 95%), namely, pairs O06239/C6DPS4 (97.87%), A5U3R8/O07733 (95.54%), A5WU15/P72030 (98.54%), C6DL40/O53355 (97.57%), C6DSD8/O06296 (98.55%), and Q7U0W0/C1ALY5 (99.72%). Another three pairs of proteins share a sequence similarity higher than 60%, i.e., C6DUE9/O06291 (68.56%), C6DWG6/O05916 (61.07%), and C6DTY5/A5TZZ2 (79.86%). We examined all of the protein pairs and distinguished two cases of homologous proteins in comparison with their corresponding unique proteins. In the first case, some N-terminal residues were missing, as well as in the second case additional N-terminal residues were contained, thereby disrupting the structure of signal peptides.

Specifically, the first case of homologous proteins includes O53355, C6DPS4, and O06296. Because some residues are missing, the n-regions before the Ile, Leucine, and Valine hydrophobic residues are shorter. Interestingly, O53355 in MYCTU is a homolog of the unique protein C6DL40 in MYCTK. Moreover, it is annotated as a high-confidence drug target in the MYCTU strain without signal peptide annotation or prediction. In contrast, the unique protein C6DL40 has more N-terminal residues, including four positive residues of arginine, which extend the n-region. This probably accounts for the signal peptide structure. The pair comprised of C6DPS4 in MYCTK and the unique protein O06239 in MYCTU is also interesting because O06239 is annotated as a multi-spanning transmembrane protein and a high-confidence drug target in UniProt. However, UniProt/Swiss-Prot does not annotate O06239 as a signal peptide protein, but SVMSignal indicates that a signal peptide exists.

The second case of homologous proteins includes O07733, P72030, O06291, O05916, and A5TZZ2. Among them, O07733, P72030 and A5TZZ2 have additional charge-intensive regions at the N-terminus, but they do not have h-region followed. The other two proteins, O06291 and O05916, have over 150 additional residues at the N-terminus, and their remaining subsequences are identical to their corresponding unique proteins. Although SVMSignal predicts that O05916 may have cleavage sites, the protein is not predicted as a signal peptide protein.

#### O06239 (Rv2136c) vs. C6DPS4 (TBMG_01845)

The unique signal peptide protein O06239 of MYCTU is encoded by Rv2136c.The Rv2136c gene encodes a putative homologue of E. coli's UppP [36]. Since Mycobacterium tuberculosis (Mtb) has the ability to block phagosome acidification and thereby contributing to resist the drug bacitracin [37], [38], researchers are interested in discovering the genes responsible for Mtb's acid resistance. Vandal et al. used transposon mutagenesis to discover genes for acid resistance, and then measuring the intrabacteria pH values of the knockout strains [37]. Initially, they took Rv2136c and Rv3671c as candidates because the mutation in these two genes would cause the strain sensitive to acid. However, they only performed further analysis on Rv3671c because mutant phenotypes of Rv2136c did not revert while the wild-type allele “in trans" was given. Later, Darby et al. deleted the Rv2136c gene in the H37Rv strain, but could not obtain any phenotype in the Rv2136c transposon mutant strain mentioned in Vandal et al.'s experiment [39]. It is mentioned in the paper that they started sequencing for the Rv2136c transposon mutant strain to verify whether acid resistance is from mutated Rv2136c or other mutated genes.

In our study, the protein O06239 contains its similar non-signal peptide protein C6DPS4 of MYCTK as a long subsequence and has additional 6 amino acids “MTAAPA" starting from the N-terminus. But these two proteins are predicted by our predictor to have dramatically different prediction results, one having signal peptide and the other none. The additional amino acids show strong amphiphilicity and the secondary structure predictor JPred 3 predicted that there is a highly probable helix structure [40]. Therefore, the N-terminal segment of O06239 may very likely form an amphipathic helix.

Furthermore, we also noticed that the Rv2136c encoded proteins have different sequence lengths, for example, NP_216652.1 (276 aa), CAB08657.1 (276 aa), O06239.2 (282 aa). These sequences are reported to have length differing at six amino acids, and such finding on Rv2136c encoded proteins seems to be controversial. Notably, the homologous proteins O06239 and C6DPS4 have lengths of 282 and 276, respectively. As Rv2136c encoded proteins are reported to have different lengths, the sequence of Rv2136c encoded proteins need to be further verified.

Furthermore, O06239 is a predicted membrane protein containing five transmembrane domains as annotated in Uniprot/Swiss-Prot and is predicted by SVMSignal to have an extensively cleavable region. If the longer sequence indeed contains a cleavable signal peptide, then this transmembrane protein will be secreted to elsewhere or remain in membrane after signal peptide cleaved. If the correct sequence is the short version, then the protein will not be secreted and forms a transmembrane protein with an N-terminal amphipathic helix. In summary, the first 100 amino acids of O06239 and C6DPS4 are different and thus result in different signal peptide prediction results. Moreover, the Rv2136c transposon mutant strain of H37Rv strain is acid resistant same as Rv3671c; however, the Rv2136c knockout strain is not sensitive to acid. Is this phenomenon related to whether the existence of signal peptide in the Rv2136c encoded sequence? The sequence of Rv2136c encoded protein needs to be further clarified.

#### A5U3R8 (MRA_1910) vs. O07733 (Rv1899c)

The non-signal peptide protein O07733 of MYCTU encoded by the gene Rv1899c was determined as a unique protein in Mycobacterium bovis BCG strain when compared with H37Rv, which was confirmed by 2-DE and mass spectrometry [41]. It is also suggested that this protein may be related to virulence attenuation [41].

Herrmann et al. [42] studied post-translationally modification, specifically glycosylation, of Mycobacterium tuberculosis proteins. They considered protein of Rv1899c as a candidate for O-glycosylation lipoprotein; however, its glycosylation was not confirmed in their experiment. Later, this protein was annotated as one of H37Rv membrane proteins as determined by one-dimensional SDS gels with LC-MS (liquid chromatography-mass spectrometry) [43]. Interestingly and contradictorily, this protein was annotated as exported lipoprotein by 2-DE combined with MALDI-TOF MS and LC-MS/MS [44]. Note that O07733 is annotated as uncharacterized protein, but annotated in GO as plasma membrane and extracellular region. It becomes a question worth of further investigation whether O07733 encoded by Rv1899c is localized at the membrane or exported extracellular.

The homologous proteins A5U3R8 (MRA_1910) and O07733 (Rv1899c) have 343 amino acids and 359 amino acids, respectively. O07733 contains A5U3R8 as a subsequence and has additional 16 amino acids at the N-terminus. Both of them have only one weak hydrophobic core near the N-terminus. If O07733 is a membrane protein, the only hydrophobic region would be the membrane core. Moreover, this hydrophobic region may form an amphipathic helix because high amphiphilicity around the region are observed from SVMSignal's visualization of biochemical profiles and the secondary structure predictor JPred 3 [40] predicts it as a helix.

Furthermore, the protein sequence of O07733 provided by UniProt/Swiss-Prot is slightly different from the protein sequence annotated for the gene Rv1899c (CAB10035.1) in the GenBank, the former 359 amino acids and the latter 343 amino acids. It is worthwhile to verify the sequence of Rv1899c protein and determine the existence of signal peptide therein. Note that NetOGlyc [45] predicts several glycosylation sites on O07733, and proteins with signal peptide are more probable to have O-glycosylation. If SVMSignal's prediction on O07733 as non-signal peptide protein is correct, it may explain why the Rv1899c protein did not occur in the experiment reported in [42] and supports the experiment in [43] that Rv1899c is a membrane protein. Its homologous protein A5U3R8 of the attenuated strain MYCTA is predicted to contain signal peptide by SVMSignal, and the existence of signal peptide may support glycosylation.

#### A5WU15 (TBFG_13829) vs. P72030 (Rv3795)

The protein P72030, also called EmbB protein, of MYCTU encoded by gene Rv3795 was a drug target. The first line drug Ethambutol (EMB) can inhibit three proteins EmbA, EmbB, and EmbC in tuberculosis. Ethambutol targeted proteins, i.e., Emb proteins, are critical to synthesis arabinogalactan which is involved in cell wall. The mutation of the embB gene can be observed in Ethambutol-resistant strain [46]. Genetic polymorphisms of embB can also be observed in several drug-resistance strains [47].

Though the structures of tuberculosis Emb membrane proteins have not been solved, the C-terminal hydrophilic domain was solved in 2011, and the finding supports the suggestion that Emb proteins function as dimers, the combination of EmbC/EmbC and EmbA/EmbB [48].

The protein A5WU15 of MYCTF is identical to P72030 except lack of the first 16 amino acids at the N-terminus. SVMSignal predicted A5WU15 as signal peptide protein due to its deletion of the 16 amino acids, making the n-region suitable for forming signal peptide. If A5WU15 really contains signal peptide, the cleaved sequence will somewhat affect the structure of EmbB through removing the first transmembrane domain and the cleaved positive residues may also affect topology.

#### C6DL40 (TBMG_03351) vs. O53355 (Rv3303c)

The protein O53355, named LpdA, of MYCYU is encoded by the Rv3303c gene. The LpdA had been thought as a probable Mtb's dehydrogenases; however, it was verified as a NAD(P)H quinone reductase [49]. The protein of Rv3303c was also supposed to contribute to the virulence because the NAD(P)H quinone reductase may remove reactive oxygene [50]. Using qRT-PCR to compare Rv3303c between H37Rv strain and the attenuated H37Ra strain, the result shows that the lpdA transcript was rarely detected in the H37Ra strain, but up-regulated significantly in the H37Rv strain [51]. Therefore, the overexpression of the Rv3303c may increase Mtb's ability against oxidative stress.

The protein structure of O53355 was solved in 2004 [49] and deposited in Protein Data Bank as 1XDI. The N-terminal region forms a part of the structure, and thus it is confirmed that this protein does not have a cleavable signal peptide. It is consistent with the prediction result of SVMSignal, which predicted O53355 having no signal peptide though containing an extensively cleavage region.

The protein O53355 and its homolog in the KZN 1435 strain, i.e., C6DL40 of MYCTK, share 471 amino acids in common. O53355 has additional 22 amino acids at C-terminus, and C6DL40 has 10 additional amino acids of “HRRRARLWAV" at the N-terminus, extending the moderate hydrophobic region and forming a possible signal peptide by the possible n-region of four arginines. If the sequence of C6DL40 is correct and the signal peptide exists, then we can suppose that LpdA of the KZN 1435 strain will be very likely secreted out and thus increase the ability against oxidative stress.

Moreover, in the sequence alignment of disulfide reductases including LpdA, only a motif “GGGPAG" near the N-terminus is observed and lies in the predicted signal peptide domain of KZN 1435 strain. Since the possible interaction residues R (245), S (213), and Q (214) of the Rv3303c protein do not lie in the signal peptide domain, cleaving signal peptide may not affect the function of quinone reductase [49].

#### C6DUE9 (TBMG_02759) vs. O06291 (Rv1223)

The Rv1223 gene encodes the protein HtrA1 in Mtb, i.e., O06291, which was reported as a single-spanning transmembrane protein [32], [52], [53]. Since this protein has only one hydrophobic region, which forms a transmembrane domain, it is consistent with SVMSignal's prediction that there is no cleavable N-terminal signal peptide.

The HtrA family proteins in Mtb include HtrA1 (encoded by Rv1223), HtrA2 (Rv0983) and HtrA3 (Rv0125); however, only HtrA2's structure has been determined at 2.0 Å, i.e., 2Z9I in the Protein Data Bank [54]. It was proved that HtrA2 of Mtb is related to virulence since deletion of the Rv0983 gene in the mouse model extends the survival [54]. However, the knockout of htrA1 from H37Rv strain cannot obtain generation, and thus htrA1 is supposed to be an essential protein in Mtb [54].

Though HtrA1 of the H37Rv stain (O06291) is different from HtrA1 (Q92743) in human, but the SPD (serine protease domain) and PDZ (protease domain z) domains in this two species are still conserved as these two domains in HtrA proteins are conserved from bacteria to mammalian [55], [56].

Interestingly, human HtrA1 which contains an insulin-like growth factor binding domain is suggested to have signal peptide, and then secreted out [55]. SVMSignal predicted the only hydrophobic region of human HtrA1 as signal peptide h-region, and also supported this argument.

In comparison with O06291, the HtrA1 sequence of KZN 1435 strain simply lacks the first 166 residues at the N-terminus, thus move the only hydrophobic region and two positive charge residues forward to an appropriate position for forming a signal peptide. If the sequence of KZN 1435 strain is correct and signal peptide exists, the HtrA1 of KZN 1435 strain may be secreted out. Moreover, some bacteria, e.g., helicobacter pylori, secrete their HtrA to extracellular, thereby increasing their virulence [55]. Since the sequence of the gene TBMG_02759 recorded in the GeneBank does not start from the standard start codon, it is worth of further investigation to verify the HtrA1 sequence of KZN 1435 strain and its possibility of having a signal peptide.

#### C6DWG6 (TBMG_03065) vs. O05916 (Rv0924c)

The Rv0924c encodes the MntH protein, i.e., O05916, in the Mtb H37Rv strain. The tuberculosis MntH is an orthologue of the Nramp protein (natural resistance associated macrophage protein), which mediates the divalent cation transportation. The pathogen manganese transporter may compete with the host. If a host has defects Nramp1, then the host is inclined to be infected. If Nramp transporter of a pathogen gets mutation, then virulence is attenuated. The Nramp proteins are conserved from bacteria to mammalian and the mutation have been shown to attenuate the virulence [57].

The protein of Rv0924c was initially thought to be a pH-dependent divalent cation transporter [58]. Later it was reported that the mntH knockout in the mouse model did not influence the virulence [59], [60]. However, Papp-Wallace and Maguire [57] suggested that the above conclusion is not yet definite and thought that there may be some other proteins, .e.g., sitABCD, that might compensate the loss of mntH [57]. The MntH protein in the Mtb H37Rv strain is a multi-spanning transmembrane protein, since the orthologue MntH protein of E. coli was determined as an eleven transmembrane-segment protein [61]. The difference of MntH sequences between KZN 1435 strain and H37Rv strain is that C6DWG6 lacks the first 166 residues from the N-terminus in O05916. If the MntH sequence of KZN 1435 strain is correct, then the structure of this transmembrane protein is definitely different from that of H37Rv, because of lacking at least four transmembrane domains. Additionally, if the signal peptide truly exists, the first hydrophobic region will further be cleaved and affect the structure, the topology of the MntH needs to be examined again.

## Discussion

### Using cleavage site information to predict signal peptides

SVMSignal first predicts the potential cleavage sites of a sequence and then predicts the signal peptide sequence. If we consider sequences that possess potential cleavage sites as signal peptides, the naïve predictor denoted as “Cleavable" in Table 3 achieves nearly perfect sensitivity on the benchmark SPDB and SP datasets, irrespective of the type of organism. It seems that the information in signal peptide cleavage sites can be learned easily; however, Cleavable predicted several false-positive signal peptides, and also failed to classify transmembrane proteins and globular proteins with poor MCCs and specificities, as shown in Table 1 and Table 4, respectively. The above observation implies that cleavage site information may be easy to learn, but it is not sufficient to characterize a signal peptide. We believe that including the structural information of signal peptides in the Cleavable predictor could improve signal peptide prediction significantly, as evidenced by SVMSignal's high sensitivity, specificity, and MCC on the benchmark datasets. In particular, the classification was improved from the Cleavable predictor by over 50% in MCC. This finding implies that learning signal peptide structures is an effective modeling approach.

### Large-scale signal peptide analysis of the HAMAP database

Bacteria are a major type of human pathogen, and their secreted proteins often affect hosts. Since the annotations of microbial proteomes in the UniProt/Swiss-Prot database are incomplete, we conducted a large-scale secreted protein analysis of the HAMAP database. We applied SVMSignal to the HAMAP database (release Feb 2011), which contains 91 archaea, 1130 bacteria, and 71 eukaryota proteomes, to predict signal peptide proteins, and then determined the secreted proteins accordingly.

SVMSignal's prediction results on the entire HAMAP database are provided in Supplementary Data at http://bio-cluster.iis.sinica.edu.tw/SVMSignal. Here, we consider some general statistics, which are detailed in Table S1. First, the average percentage of signal peptide proteins in each proteome is 8.13% in the archaea, 13.34% in the bacteria, and 10.07% in the eukaryotes. Second, in the archaea, Halalkalicoccus jeotgali (strain DSM 18796/CECT 7217/JCM 14584/KCTC 4019/B3) has the lowest percentage of signal peptide proteins (4.63%), and Methanoplanus petrolearius (strain DSM 11571/OCM 486/SEBR 4847) has the highest percentage (14.32%). In the bacteria, Zinderia insecticola (strain CARI) has 0%, i.e., no signal peptide proteins, and Bdellovibrio bacteriovorus has the highest percentage (32.51%). In the eukaryotes, Hemiselmis andersenii has the lowest percentage (0.80%), and Pediculus humanus subsp. corporis has the highest percentage (26.76%.) Finally, the average length of signal peptides is 26.56 residues in the archaea, 26.02 residues in the bacteria, and 23.41 residues in the eukaryota.

### Transmembrane proteins with signal peptides in HAMAP proteomes

We also examined transmembrane proteins with signal peptides in all microbial proteomes of HAMAP because some crystallized transmembrane proteins contain signal peptides that are easily confused with transmembrane domains. We removed signal peptides from the proteins and used TMHMM [34], [35] to predict whether the cleaved proteins contained transmembrane domains. The average percentage of transmembrane proteins with signal peptides in the archaea, bacteria and eukaryotes are 3.14%, 2.62% and 2.10%, respectively. In the archaea, Methanosphaera stadtmanae (strain DSM 3091) has the lowest percentage (1.37%), and Aciduliprofundum boonei (strain DSM 19572/T469) has the highest percentage (4.74%). In the bacteria, Carsonella ruddii (strain PV) and Zinderia insecticola (strain CARI) have 0% and Arcobacter nitrofigilis (strain ATCC 33309/DSM 7299/LMG 7604/NCTC 12251/CI) has the highest percentage (5.76%). In the eukaryotes, Hemiselmis andersenii has 0%, and Caenorhabditis elegans has the highest percentage (4.62%).

### Web service

SVMSignal provides a web service that accepts queries with up to 10,000 sequences in FASTA format. As well as the prediction results, SVMSignal provides the calculated biochemical properties, including the free energy, polarity, average volume and charge index of each residue. The properties are presented in a compact profile graph for visualization to help users examine potential signal peptide structures, such as the n-region, h-region, and c-region. Users can download all the information in the first 100 residues, including predictions in FASTA format, all the profile values in tab-delimited text format, and the graphs of the profiles.

## Materials and Methods

### SVMSignal-a hierarchical SVM-based predictor

**Figure 1:**
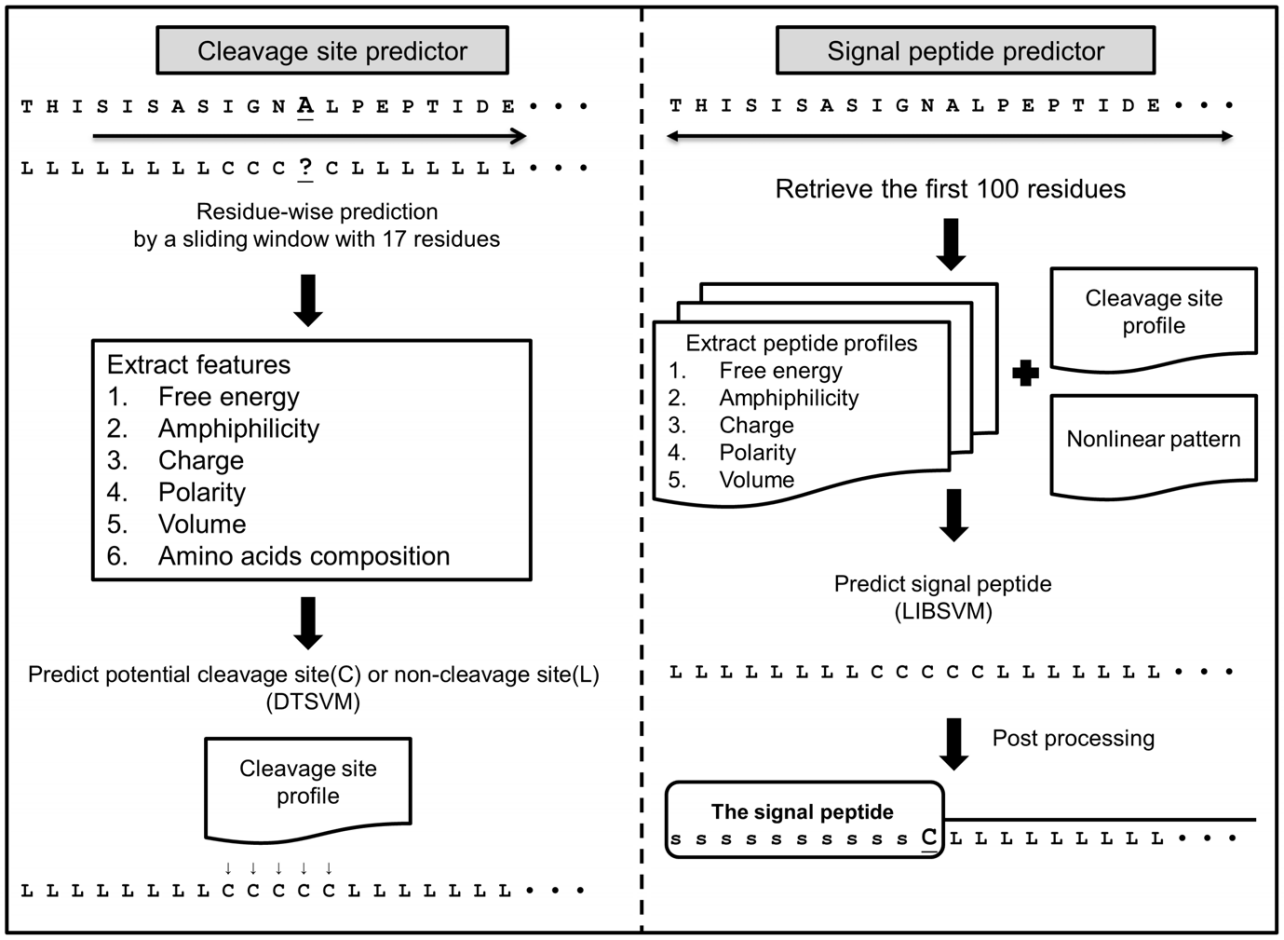
The hierarchical architecture of SVMSignal.

SVMSignal performs signal peptide prediction in two stages using support vector machines (SVMs) as classifiers, as illustrated in Figure 1. It predicts signal peptides from the first 100 residues of each protein sequence. The first SVM classifies every residue into around the cleavage site, denoted by “C", or outside the cleavage site, denoted by “L." Since protein sequences containing signal peptides have cleavage sites, the results from the first classifier can help the user determine whether a protein sequence contains a signal peptide. The second classifier integrates the predictions of the first classifier and other features to classify each residue into the signal peptide region denoted by “s" or the non-signal peptide region denoted by “L." Note that the last predicted residue of the signal peptide is denoted by “C." If a protein sequence does not contain any signal peptides, all of the 100 residues will be assigned the label “L".

More specifically, the first classifier is a residue-wise predictor. Given a protein sequence, the residue to be predicted is centered in a window of length 17. The features derived from a 17-mer sequence for prediction include: (i) position-specific free energy of transmembrane helix insertion [62] to describe the hydrophobic core, called free energy for convenience; (ii) amphiphilicity [63]; (iii) charge index [64]; (iv) polarity [65]; (v) residue volume index [66], and (vi) amino acid composition. For the first and last eight residues in the first classifier, we use small values to complete the columns corresponding to non-existing residues. The second classifier is a chain-wise predictor that uses the following features: (i) the prediction results from the first classifier; (ii) the first five feature profiles with a dimension of 100 used in the first predictor; and (iii) a novel nonlinear pattern feature that captures the relationships between any two of the five feature profiles, e.g., the sequence distance between positively charged residues and the hydrophobic region. All of the features are normalized to a [0, 1] closed interval.

### Biochemical features

The five features used in the both predictors are described below. The free energy profile, amphiphilicity profile and polarity profile are normalized, respectively, by the sigmoidal functions given by 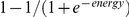, 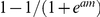 and 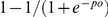, where “*energy*" denotes the free energy of transmembrane helix insertion estimated by Hessa et al.'s method [62]; “*am*" denotes the amphiphilicity derived by Mitaku et al.'s method [63]; and “*po*" denotes the mean residue polarity calculated by Radzicka and Wolfenden's method [65]. The charge profile is obtained by defining positively charged residues as 1, neutral residues as 0.5, and negatively charged residues as 0 based on the index used by Klein et al. [64]. The volume profile is used by Pontius et al. [66] and normalized by dividing the maximum volume value 237.2.

To determine the amino acid composition of each window, we use the natural language processing method described in Leopold and Kindermann [67]. A protein subsequence of length *l* can be treated as a document, which is a vector containing twenty types of words, corresponding to amino acids. First, we calculate the frequencies of the amino acids in each document in the training data. Let the word *w*_k_ be the *k*th type of amino acid. In addition, let *k*=1, 2 … 20, 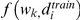 be the frequency of *w*_k_ in document *i*; and let 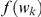 denote the frequency of *w*_k_ in all *N* documents in the training data, i.e., 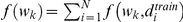. Second, we calculate the importance weights of the amino acids, denoted by 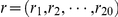 in all the documents in the training data by the equation 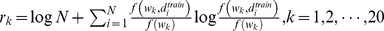. The amino acid composition feature is given by the component-wise product of 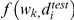 and *r*, and then normalized by the *L*_2_ norm.

A feature value close to 1 means the corresponding residue is more hydrophobic and more amphiphilic, is positively charged, and has a larger volume and a higher polarity. In the first classifier, we fill 0.5 as features for nonexistent residues in windows centered by residues of the N-terminus and C-terminus for all profiles, except charge and volume, we fill zero.

**Figure 2:**
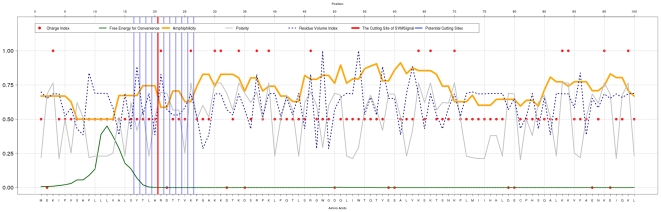
An example, O95994 (AGR2_HUMAN), of biochemical feature profiles in a 100-residue N-terminal subsequence predicted to contain a signal peptide.

SVMSignal displays the feature profiles on the web service. Figure 2 shows an example of feature profiles. The green curve denotes the free energy, the red dots are charge indices; the gray curve represents the polarity; the blue dotted curve indicates the residue volumes; the orange curve represents the amphiphilicity; the blue vertical lines are predicted *potential* cleavage sites; and the red vertical line is the predicted cleavage site after post-processing. The visualization of biochemical feature profiles helps users recognize signal peptide structures. This example shows a clear signal peptide structure, which is given by the charged residues followed by the hydrophobic core and then by the c-region containing the small neutral and polar residues. Furthermore, there are several potential cleavage sites after the hydrophobic core, but only one of them lies near more polar and small residues.

### Novel profile pattern features used to capture the relationship between two biochemical profiles

Given any two of the above five biochemical features, we can define a nonlinear profile pattern feature to capture the relationship between two profiles by determining the distance of a shift between the profiles' peaks. For example, to model the signal peptide structure of charged residues in the n-region followed by the hydrophobic residues in the h-region, we shift the charge profile by some amino acids (i.e., distance) and compute the dot product of the free energy profile and the shifted charge profile. A larger dot product implies a better match between the profile peaks, and the shift distance determines the sequential relationship. If maximizing the above dot product yields a positive shift, it implies that the charged residues are followed by a hydrophobic core.

Specifically, let *f* be a vector corresponding to a feature profile, where *f*(*i*) is the feature value of the *i*th residue for *i*=1,2,…,100; and *f*(*i*) is 0 otherwise. Similarly, let *g* be a vector corresponding to another feature profile. We determine the best shift *S*_1_ by the maximum dot product derived by Equation (1). To capture a profile's skewness near the beginning and end positions, i.e., positions 1 and 100, we reverse the profile *g* and then find the best shift S_2_, as shown in Equation (2).
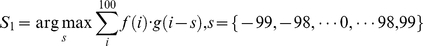
(1)

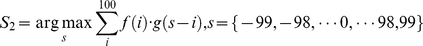
(2)Furthermore, we normalize the nonlinear features *S*_1_ and *S*_2_ extracted from the *f* and *g* profiles by simply using the range of possible shifts, i.e., normalized 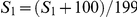. In this way, we calculate twenty nonlinear pattern features for all permutations of the five biochemical profiles.

### Post-processing to determine cleavage sites

Potential cleavage sites are determined according to the residues predicted with “C" labels by the first classifier. Since the first classifier performs residue-wise prediction, the residues labeled with “C" may occur sparsely anywhere in the query. First, we identify the ten-residue region nearest the N-terminus that contains the most “C" labels. We consider ten-residue regions because we defined five residues around the cleavage site as positive data. Next, we determine the position of the last residue in the signal peptide from the residues with “C" labels in the region that minimizes the total distance between the positions of all “C" labels.

### Training the SVM predictors

As the first predictor of SVMSignal is residue-wise and needs to handle a large number of unstable training samples, we use DTSVM [68] to train the first classifier. In the training stage, for each sequence containing a signal peptide, we label the five residues before and after the cleavage site, a total of 10 residues, as “C" for positive data and “L" for negative data because residues near the cleavage site may have similar biochemical properties. DTSVM requires both training and validation sets, so we divide the Phobius 2004 dataset [16] (described later) into 80% for training, 10% for validation, and 10% for cross-validation. The second classifier is a chain-wise predictor and contains 2654 protein chains for training. Because the sample size is not large, we use LIBSVM [69] with an RBF kernel function to train the model directly. Then, we integrate the two classifiers to perform 10-fold cross-validation on the Phobius dataset to determine the parameters “cost" (2^1.5^=2.8284) and “gamma" (2^−4.5^=0.0442) in the kernel function of LIBSVM.

### Training dataset

To avoid over-estimating our predictor's performance, we use the Phobius dataset [16] to train SVMSignal. The dataset contains 247 transmembrane protein sequences, 45 transmembrane protein sequences with signal peptides, 1,275 sequences containing signal peptides without transmembrane domains and 1,087 protein sequences that do not have signal or transmembrane domains.

### Benchmark datasets

#### SPdb dataset

We downloaded SPdb (release 5.1) [19], a signal peptide database containing signal sequences of archaea, bacteria, viruses and eukaryotes with all the sequences derived from Swiss-Prot (release 55.0 2008). Then, taking all 2,512 experimentally verified signal peptide sequences in the dataset, we used CD-HIT [33] to filter out sequences whose first 100 residues shared at least 30% sequence similarity with sequences in the Phobius training dataset. The process produced a dataset of 656 sequences, which we call the SPDB dataset.

#### UniProt/Swiss-Prot and PDBTM datasets

To evaluate the predictors' ability to distinguish signal sequences from non-signal sequences, we compiled a dataset from UniProt/Swiss-Prot (downloaded in December 2010) and PDBTM [21] (downloaded on November 5, 2010). We divided the dataset into three disjoint groups, i.e., sequences containing signal peptides (denoted by SP), sequences containing only transmembrane domains (denoted by TM), and sequences that did not contain transmembrane domains or signal peptides (denoted by G). The SP dataset was obtained by querying signal peptides in UniProt/Swiss-Prot with the evidence code “experiment" or “probable". Note that the SP dataset includes some signal peptides with “Transmembrane [KW-0812]" or “Membrane [KW-0472]" annotations. The TM dataset was obtained by querying “Transmembrane" annotated with “experiment" or “probable" evidence codes in UniProt/Swiss-Prot and removing any data with the signal peptide annotation in any evidence code. We further enlarged the TM dataset by extracting sequences in PDBTM with structures solved by X-rays with a resolution less than 4.0 Å. The G dataset contained sequences without signal peptides or TM annotations. Finally, for each dataset we filtered out sequences whose first 100 residues share at least 30% similarity with sequences in the Phobius training dataset by using CD-HIT. The resulting SP, TM, and G datasets contained 901, 104, and 5,755 sequences, respectively.

## Supporting Information

Text S1 **The alignments of unique proteins and their homologous proteins.**
(TXT)

Dataset S1 **Accession IDs of the benchmark datasets.**
(XLS)

Dataset S2 **The results of SVMSignal prediction on the seven selected tuberculosis strains.**
(ZIP)

Dataset S3 **Accession IDs of proteins predicted as signal peptide proteins in the seven selected tuberculosis strains.**
(XLS)

Table S1 **General analysis of proteomes in the HAMAP database.**
(XLS)

## References

[1] Jarjanazi H, Savas S, Pabalan N, Dennis JW, Ozcelik H (2008). Biological implications of SNPs in signal peptide domains of human proteins.. Proteins: Structure, Function and Genetics.

[2] Grønborg M, Kristiansen TZ, Iwahori A, Chang R, Reddy R (2006). Biomarker discovery from pancreatic cancer secretome using a differential proteomic approach.. Molecular and Cellular Proteomics.

[3] Piersma SR, Fiedler U, Span S, Lingnau A, Pham TV (2010). Workflow comparison for label-free, quantitative secretome proteomics for cancer biomarker discovery: Method evaluation, differential analysis, and verification in serum.. Journal of Proteome Research.

[4] Kulasingam V, Diamandis EP (2008). Strategies for discovering novel cancer biomarkers through utilization of emerging technologies.. Nature Clinical Practice Oncology.

[5] Paetzel M, Karla A, Strynadka NCJ, Dalbey RE (2002). Signal peptidases.. Chemical Reviews.

[6] Von Heijne G (1990). The signal peptide.. Journal of Membrane Biology.

[7] Gierasch LM (1989). Signal sequences.. Biochemistry.

[8] Andersson H, Von Heijne G (1991). A 30-residue-long ‘export initiation domain’ adjacent to the signal sequence is critical for protein translocation across the inner membrane of Escherichia coli.. Proceedings of the National Academy of Sciences of the United States of America.

[9] Hikita C, Mizushima S (1992). Effects of total hydrophobicity and length of the hydrophobic domain of a signal peptide on in vitro translocation efficiency.. Journal of Biological Chemistry.

[10] Nielsen H, Engelbrecht J, Von Heijne G, Brunak S (1996). Defining a similarity threshold for a functional protein sequence pattern: The signal peptide cleavage site.. Proteins: Structure, Function and Genetics.

[11] von Heijne G (1983). Patterns of amino acids near signal-sequence cleavage sites.. European Journal of Biochemistry.

[12] Nielsen H, Engelbrecht J, Brunak S, Von Heijne G (1997). Identification of prokaryotic and eukaryotic signal peptides and prediction of their cleavage sites.. Protein Engineering.

[13] Nielsen H, Krogh A (1998). Prediction of signal peptides and signal anchors by a hidden Markov model.. Proc Int Conf Intell Syst Mol Biol.

[14] Bendtsen JD, Nielsen H, Von Heijne G, Brunak S (2004). Improved prediction of signal peptides: SignalP 3.0.. Journal of Molecular Biology.

[15] Hiller K, Grote A, Scheer M, Münch R, Jahn D (2004). PrediSi: Prediction of signal peptides and their cleavage positions.. Nucleic Acids Research.

[16] Käll L, Krogh A, Sonnhammer ELL (2004). A combined transmembrane topology and signal peptide prediction method.. Journal of Molecular Biology.

[17] Plewczynski D, Slabinski L, Tkacz A, Kajan L, Holm L (2007). The RPSP: Web server for prediction of signal peptides.. Polymer.

[18] Reynolds SM, Käll L, Riffle ME, Bilmes JA, Noble WS (2008). Transmembrane topology and signal peptide prediction using dynamic Bayesian networks.. PLoS Computational Biology.

[19] Choo KH, Tan TW, Ranganathan S (2005). SPdb - A signal peptide database.. Bmc Bioinformatics.

[20] Wu CH, Apweiler R, Bairoch A, Natale DA, Barker WC (2006). The Universal Protein Resource (UniProt): an expanding universe of protein information.. Nucleic acids research.

[21] Tusnády GE, Dosztányi Z, Simon I (2004). Transmembrane proteins in the Protein Data Bank: Identification and classification.. Bioinformatics.

[22] Ramamurthi KS, Schneewind O (2002). Type III protein secretion in Yersinia species..

[23] Christie PJ, Atmakuri K, Krishnamoorthy V, Jakubowski S, Cascales E (2005). Biogenesis, architecture, and function of bacterial type IV secretion systems..

[24] Chang YH, Wu CC, Chang KP, Yu JS, Chang YC (2009). Cell secretome analysis using hollow fiber culture system leads to the discovery of CLIC1 protein as a novel plasma marker for nasopharyngeal carcinoma.. Journal of Proteome Research.

[25] Luo X, Liu Y, Wang R, Hu H, Zeng R (2011). A high-quality secretome of A549 cells aided the discovery of C4b-binding protein as a novel serum biomarker for non-small cell lung cancer.. Journal of Proteomics.

[26] Lima T, Auchincloss AH, Coudert E, Keller G, Michoud K (2009). HAMAP: A database of completely sequenced microbial proteome sets and manually curated microbial protein families in UniProtKB/Swiss-Prot.. Nucleic Acids Research.

[27] Pallen MJ, Wren BW (2007). Bacterial pathogenomics.. Nature.

[28] Tseng TT, Tyler BM, Setubal JC (2009). Protein secretion systems in bacterial-host associations, and their description in the Gene Ontology.. BMC Microbiology.

[29] Walzl G, Ronacher K, Hanekom W, Scriba TJ, Zumla A (2011). Immunological biomarkers of tuberculosis.. Nature Reviews Immunology.

[30] Banaiee N, Kincaid EZ, Buchwald U, Jacobs WR, Ernst JD (2006). Potent inhibition of macrophage responses to IFN-γ by live virulent Mycobacterium tuberculosis is independent of mature mycobacterial lipoproteins but dependent on TLR2.. Journal of Immunology.

[31] McDonough JA, McCann JR, Tekippe EM, Silverman JS, Rigel NW (2008). Identification of functional Tat signal sequences in Mycobacterium tuberculosis proteins.. Journal of Bacteriology.

[32] Målen H, Pathak S, Søfteland T, De Souza GA, Wiker HG (2010). Definition of novel cell envelope associated proteins in Triton X-114 extracts of Mycobacterium tuberculosis H37Rv.. BMC Microbiology.

[33] Li W, Godzik A (2006). Cd-hit: A fast program for clustering and comparing large sets of protein or nucleotide sequences.. Bioinformatics.

[34] Sonnhammer EL, von Heijne G, Krogh A (1998). A hidden Markov model for predicting transmembrane helices in protein sequences.. Proc Int Conf Intell Syst Mol Biol.

[35] Krogh A, Larsson B, Von Heijne G, Sonnhammer ELL (2001). Predicting transmembrane protein topology with a hidden Markov model: Application to complete genomes.. Journal of Molecular Biology.

[36] Cole ST, Brosch R, Parkhill J, Garnier T, Churcher C (1998). Deciphering the biology of mycobacterium tuberculosis from the complete genome sequence.. Nature.

[37] Vandal OH, Pierini LM, Schnappinger D, Nathan CF, Ehrt S (2008). A membrane protein preserves intrabacterial pH in intraphagosomal Mycobacterium tuberculosis.. Nature Medicine.

[38] MacMicking JD, Taylor GA, McKinney JD (2003). Immune Control of Tuberculosis by IFN-γ-inducible LRG-47.. Science.

[39] Darby CM, Venugopal A, Ehrt S, Nathan CF (2011). Mycobacterium tuberculosis gene Rv2136c is dispensable for acid resistance and virulence in mice.. Tuberculosis.

[40] Cole C, Barber JD, Barton GJ (2008). The Jpred 3 secondary structure prediction server.. Nucleic Acids Research.

[41] Büttner K, Bernhardt J, Scharf C, Schmid R, Mäder U (2001). Identification of proteins Mycobacterium tuberculosis missing in attenuated Mycobacterium bovis BCG strains.. Electrophoresis.

[42] Herrmann JL, Delahay R, Gallagher A, Robertson B, Young D (2000). Analysis of post-translational modification of mycobacterial proteins using a cassette expression system.. Febs Letters.

[43] Gu S, Chen J, Dobos KM, Bradbury EM, Belisle JT (2003). Comprehensive proteomic profiling of the membrane constituents of a Mycobacterium tuberculosis strain.. Molecular & cellular proteomics : MCP.

[44] Målen H, Berven FS, Fladmark KE, Wiker HG (2007). Comprehensive analysis of exported proteins from Mycobacterium tuberculosis H37Rv.. Proteomics.

[45] Julenius K, Mølgaard A, Gupta R, Brunak S (2005). Prediction, conservation analysis, and structural characterization of mammalian mucin-type O-glycosylation sites.. Glycobiology.

[46] Sreevatsan S, Stockbauer KE, Pan X, Kreiswirth BN, Moghazeh SL (1997). Ethambutol resistance in Mycobacterium tuberculosis: Critical role of embB mutations.. Antimicrobial Agents and Chemotherapy.

[47] Loerger TR, Koo S, No EG, Chen X, Larsen MH (2009). Genome analysis of multi- and extensively-drug-resistant tuberculosis from KwaZulu-Natal, South Africa.. Plos One.

[48] Alderwick LJ, Lloyd GS, Ghadbane H, May JW, Bhatt A (2011). The C-terminal domain of the arabinosyltransferase mycobacterium tuberculosis EmbC is a lectin-like carbohydrate binding module.. PLoS Pathogens.

[49] Argyrou A, Vetting MW, Blanchard JS (2004). Characterization of a new member of the flavoprotein disulfide reductase family of enzymes from Mycobacterium tuberculosis.. Journal of Biological Chemistry.

[50] Akhtar P, Srivastava S, Srivastava A, Srivastava M, Srivastava BS (2006). Rv3303c of Mycobacterium tuberculosis protects tubercle bacilli against oxidative stress in vivo and contributes to virulence in mice.. Microbes and Infection.

[51] Zheng H, Lu L, Wang B, Pu S, Zhang X (2008). Genetic basis of virulence attenuation revealed by comparative genomic analysis of Mycobacterium tuberculosis strain H37Ra versus H37Rv.. Plos One.

[52] Xiong Y, Chalmers MJ, Gao FP, Cross TA, Marshall AG (2005). Identification of Mycobacterium tuberculosis H37Rv integral membrane proteins by one-dimensional gel electrophoresis and liquid chromatography electrospray ionization tandem mass spectrometry.. Journal of Proteome Research.

[53] Mattow J, Siejak F, Hagens K, Schmidt F, Koehler C (2007). An improved strategy for selective and efficient enrichment of integral plasma membrane proteins of mycobacteria.. Proteomics.

[54] MohamedMohaideen NN, Palaninathan SK, Morin PM, Williams BJ, Braunstein M (2008). Structure and function of the virulence-associated high-temperature requirement A of Mycobacterium tuberculosis.. Biochemistry.

[55] Clausen T, Kaiser M, Huber R, Ehrmann M (2011). HTRA proteases: Regulated proteolysis in protein quality control.. Nature Reviews Molecular Cell Biology.

[56] Singh N, Kuppili RR, Bose K (2011). The structural basis of mode of activation and functional diversity: A case study with HtrA family of serine proteases.. Archives of Biochemistry and Biophysics.

[57] Papp-Wallace KM, Maguire ME, Ornston LN, Balows A, Gottesman S, Harwood CS (2006). Manganese transport and the role of manganese in virulence..

[58] Agranoff D, Monahan IM, Mangan JA, Butcher PD, Krishna S (1999). Mycobacterium tuberculosis expresses a novel pH-dependent divalent cation transporter belonging to the Nramp family.. Journal of Experimental Medicine.

[59] Boechat N, Lagier-Roger B, Petit S, Bordat Y, Rauzier J (2002). Disruption of the gene homologous to mammalian Nramp1 in Mycobacterium tuberculosis does not affect virulence in mice.. Infection and Immunity.

[60] Domenech P, Pym AS, Cellier M, Barry CE, Cole ST (2002). Inactivation of the Mycobacterium tuberculosis Nramp orthologue (mntH) does not affect virulence in a mouse model of tuberculosis.. FEMS Microbiology Letters.

[61] Courville P, Chaloupka R, Veyrier F, Cellier MFM (2004). Determination of Transmembrane Topology of the Escherichia coli Natural Resistance-associated Macrophage Protein (Nramp) Ortholog.. Journal of Biological Chemistry.

[62] Hessa T, Meindl-Beinker NM, Bernsel A, Kim H, Sato Y (2007). Molecular code for transmembrane-helix recognition by the Sec61 translocon.. Nature.

[63] Mitaku S, Hirokawa T, Tsuji T (2002). Amphiphilicity index index of polar amino acids as an aid in the characterization of amino acid preference at membrane-water interfaces.. Bioinformatics.

[64] Klein P, Kanehisa M, DeLisi C (1984). Prediction of protein function from sequence properties. Discriminant analysis of a data base.. Biochimica et Biophysica Acta (BBA)/Protein Structure and Molecular.

[65] Radzicka A, Wolfenden R (1988). Comparing the polarities of the amino acids: Side-chain distribution coefficients between the vapor phase, cyclohexane, 1-octanol, and neutral aqueous solution.. Biochemistry.

[66] Pontius J, Richelle J, Wodak SJ (1996). Deviations from standard atomic volumes as a quality measure for protein crystal structures.. Journal of Molecular Biology.

[67] Leopold E, Kindermann J (2002). Text categorization with support vector machines. How to represent texts in input space?. Machine Learning.

[68] Chang F, Guo CY, Lin XR, Lu CJ (2010). Tree decomposition for large-scale SVM problems.. Journal of Machine Learning Research.

[69] Chang CC, Lin CJ (2011). LIBSVM: A Library for support vector machines.. ACM Transactions on Intelligent Systems and Technology.

